# Exploring Cognitive, Behavioral, and Psychological Dimensions in Persistent Idiopathic Facial Pain and Other Chronic Orofacial Pain Conditions

**DOI:** 10.1002/brb3.71283

**Published:** 2026-03-31

**Authors:** Alessandra Telesca, Danilo Antonio Montisano, Susanna Usai, Veronica Faltracco, Alessia Ferrario, Giulia Gandini, Licia Grazzi, Giuseppe Lauria Pinter, Monica Consonni

**Affiliations:** ^1^ Neuroalgology Unit Fondazione IRCCS Istituto Neurologico Carlo Besta Milan Italy; ^2^ Neuroanestesia e Rianimazione Fondazione IRCCS Istituto Neurologico Carlo Besta Milan Italy; ^3^ SSD Centro Cefalee Fondazione IRCCS Istituto Neurologico Carlo Besta Milan Italy; ^4^ Medical Biotechnology and Translational Medicine University of Milan Milan Italy

**Keywords:** chronic pain, cognitive functioning, facial pain, headache, persistent idiopathic facial pain, persistent orofacial pain, psychological functioning, trigeminal neuralgia

## Abstract

**Background:**

Chronic orofacial pain (COP) is a complex condition often resistant to treatment and associated with psychological comorbidities. Yet, its neuropsychological profile remains under‐investigated. This case‐control study aims to identify the cognitive, behavioral, and psychological profiles of COP and their associations with clinical symptoms, with a focus on persistent idiopathic facial pain (PIFP), a condition particularly underexplored.

**Methods:**

A cohort of 42 patients (COPc), including 23 with PIFP, and 42 healthy controls (HCs) underwent a comprehensive assessment of mood, coping strategies, personality traits, cognitive functioning, and social well‐being. Between‐group and correlation analyses were performed, and Bonferroni correction was applied to account for multiple comparisons.

**Results:**

The psychological framework of COPc was marked by depressive symptoms, loneliness, alexithymia, poor quality of life, and low physical and mental well‐being. Personality assessment indicated worthlessness. Catastrophizing was a dominant coping strategy, characterized by helplessness and rumination. Cognitive assessments revealed deficits in attention and executive functions. PIFP patients exhibited particularly psychological vulnerabilities, namely, catastrophizing thinking and difficulties in describing their own feelings. Correlation analyses showed complex relationships between cognitive, behavioral, and psychological impairments in COPc, and a strong association between the negative impact of pain symptoms on social life and psychological, catastrophizing, and cognitive functioning.

**Conclusions:**

This is the first study to characterize the neuropsychological profile of PIFP and COP conditions, revealing a complex interplay of cognitive, behavioral, and psychological vulnerabilities. These findings underscore the importance of addressing both neuropsychological and social functioning in the management of chronic pain to improve patient well‐being.

## Graphical abstract

This case‐control study assessed 42 chronic orofacial pain patients, including 23 patients with persistent idiopathic facial pain, and 42 controls. COPc patients showed cognitive deficits, mood symptoms, alexithymia, and maladaptive coping, especially catastrophizing. Pain's social impact correlated with psychological and cognitive dysfunction. Findings support a biopsychosocial approach to improve care and quality of life in COP.

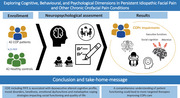

## 1 Introduction

Chronic pain affects 20% of the European population with high societal costs (Todd et al. 2019). Chronic orofacial pain (COP) is one of the most common types, recently classified as primary or secondary in ICD‐11 (Mueller et al. 2011; Treede et al. 2019). It includes various head‐related disorders, such as headaches, trigeminal neuralgia, temporomandibular disorders, and idiopathic syndromes like persistent idiopathic facial pain or burning mouth syndrome.

Diagnosis is challenging due to clinical variability, limited specialist knowledge, and heterogeneous treatment responses. Symptoms include allodynia and hyperalgesia, often associated with psychological comorbidities (anxiety, depression) and widespread pain in other body areas. Pathological mechanisms may involve peripheral or central phenomena (Shinoda et al. 2019). Non‐idiopathic facial pain may result from injuries or inflammation, increasing peripheral neuron sensitivity and activating glial cells (Sessle 2021). In idiopathic conditions, the current hypothesis is that neuroplastic changes along the pain transmission pathway may contribute to heightened sensitivity, a phenomenon known as central sensitization (Woolf 2011). Clinical manifestations of central sensitization include spontaneous pain with nuanced dermatomeric localization, source, or triggering factors, hyperalgesia exacerbated by repeated exposure, and mechanical or thermal allodynia (Harte et al. 2018; Latremoliere and Woolf 2009; Woolf 2018). Along with these clinical presentations, central sensitization is also associated with changes in sleep, mood, such as anxiety and depression, and pain catastrophizing, which are crucial aspects of patients with chronic facial pain (de Tommaso et al. 2017; Garrigós‐Pedrón et al. 2022; Li et al. 2022).

Central sensitization in orofacial pain often complicates treatment, requiring multidisciplinary care and centrally acting pharmacotherapies for effective management (Schiffman and Ohrbach 2016).

Recent studies highlight cognitive difficulties in COP patients, including attention, executive functions, and social cognition (Begasse de Dhaem and Robbins 2022; Coats et al. 2020; Telesca, Vergallito et al. 2024; Vuralli et al. 2018), along with maladaptive coping strategies and psychological profiles (Grazzi et al. 2024; Greenberg et al. 2022; Gustin et al. 2016, 2011; Telesca et al. 2023). This is in line with the literature on chronic pain diseases, where pain symptoms are considered exacerbating factors in neuropsychological alterations of patients (Chaves et al. 2021; Grabli et al. 2022; Indart et al. 2017; Jacobsen et al. 2021; Ojeda et al. 2017). However, some studies suggest that medications and comorbidities do not fully account for the observed cognitive impairment (Vuralli et al. 2018). As a result, conclusions regarding the neuropsychological functioning of COP patients remain uncertain, and the available evidence is fragmented. Most studies focus on a single condition or domain, while persistent idiopathic facial pain (PIFP), in particular, appears to be an underexplored and challenging condition to diagnose (Zakrzewska 2016).

Hence, the first aim of this study was to explore the neuropsychological profile of COP using a comprehensive approach that included cognitive, emotional, and behavioral variables, along with a specific investigation of possible interrelationships among these variables, irrespective of the pathological mechanisms underlying the diagnosis. The second aim was to identify the neuropsychological profile of patients with PIFP (Benoliel and Gaul 2017; Zakrzewska 2016), an enigmatic and under‐investigated condition frequently referred to our center, through a detailed assessment of their cognitive and psychological functioning compared with age and sex‐matched healthy controls.

Given the scarcity of data on interactions between cognitive performance and psychopathological characteristics, we conducted correlation analyses to explore potential associations among cognitive, psychological, behavioral, and personality profiles, and their relationships with clinical symptoms and pain perception.

Based on prior evidence, we hypothesized that patients suffering from PIFP and other COP conditions would exhibit reduced performance in attention, executive functions, and social cognition, as well as higher psychological distress characterized by emotional vulnerability, maladaptive coping, and personality traits that may exacerbate pain perception and functional impairment, as found in previous studies (Begasse de Dhaem and Robbins 2022; Coats et al. 2020; Fillingim et al. 2011; Greenberg et al. 2022; Jacobsen et al. 2021; Staniszewski et al. 2023; Telesca et al. 2024b). We further anticipated significant associations between cognitive and emotional–behavioral measures, consistent with the hypothesis of a functional interplay between emotional and neuropsychological domains.

## 2 Material and Methods

### 2.1 Participants

This was a mono‐centric case‐control study. Forty‐two patients with different COP conditions were enrolled as a consecutively referred clinical sample from January 2021 until January 2022, at the Neuroalgology Unit, Fondazione IRCCS Istituto Neurologico Carlo Besta in Milan, Italy. The inclusion criteria for patients were a primary diagnosis of COP according to the established criteria of ICD‐11^th^ for primary and secondary orofacial pain diseases (Benoliel et al. 2019; Nicholas et al. 2019). Each patient was examined by expert neurologists (L.G., S.U., D.A.M.) in the research group, and a full pain history, including location, etiology, and medication, was taken.

The control group consisted of 42 healthy subjects (HCs), matched for age, sex, and education, who were recruited from the regional community as well as from administrative staff members of our Institute. The criterion for inclusion in the HC group was not suffering from any chronic pain condition.

The exclusion criteria for all patients and HCs included the presence of comorbidities or neurological conditions that could affect cognition (e.g., dementia, head trauma, hydrocephalus, vascular disease), drug or alcohol abuse, neurocognitive developmental disorders, primary major psychiatric disorders (e.g., bipolar disorder, major depression), and a native language other than Italian.

The study was approved by the local ethical committee, and informed consent was obtained from all subjects. No part of the study procedures or analysis plan was pre‐registered prior to the research being conducted.

### 2.2 Procedure

After signing the written informed consent, all participants underwent a psychological interview and neuropsychological assessment with expert neuropsychologists (A.T., V.F., G.G.). The neuropsychological assessment was similar for patients and HCs. In cases of specific pain‐related questionnaires, we included specifications for HCs asking them to answer referring to sporadic pain experiences, such as episodic headache, toothache, joint or muscle pain, and any other painful disorder they have experienced. The average time calculated for completing the entire set of questionnaires was approximately half an hour, while for cognitive evaluation, when performed, lasted about 45 min. All questionnaire assessments were conducted under the supervision of experimenters.

Legal copyright restrictions prevent public archiving of the neuropsychological test battery, self‐report measures and clinical scales, which can be obtained from the copyright holders in the cited references.

#### 2.2.1 Clinical Assessment

At the time of enrolment, all patients underwent a thorough neurological examination and a clinical interview with specialists, during which the following clinical information was collected: the occurrence of any previous psychiatric conditions, assessed by asking participants whether they had ever been diagnosed with a psychiatric disorder and whether they had received pharmacological or non‐pharmacological treatment; the onset of duration of the disease (in months); sleep impairment, assessed via a single‐item question from the structured clinical interview; previous pharmacological treatments (drug classes, titrations, and duration of intake); medications being taken at the time of evaluation. Finally, the Numerical Rating Scale (NRS) for current pain was used as a unidimensional measure of pain intensity, ranging from 0 (“no pain”) to 10 (“the worst imaginable pain”), and two questions assessing the impact of symptoms on social and work life, each rated on a 0–5 Likert scale, were also administered.

#### 2.2.2 Psychological Assessment

Self‐administered scales were used to detect the behavioral and psychological functioning of patients and controls. Anxiety and depression symptoms were measured by the Hospital Anxiety Depression Scale (HADS‐A, HADS‐D) (Costantini et al. 1999). To further investigate depression, we also administered the Beck Depression Inventory—II (BDI‐II) (Beck et al. 1987; Sica and Ghisi 2007). Quality of life and the mental and psychological perceived well‐being were assessed using the Eurohis‐QoL 8‐item Index (Schiavolin et al. 2015) and the 12‐Item Short Form Survey (SF‐12) (Kodraliu et al. 2001). Additionally, we measured alexithymia (i.e., the inability to recognize or describe one's own emotions) with the Toronto Alexithymia Scale (TAS‐20) (Bressi et al. 1996), which is divided into three sub‐factors: difficulty identifying feelings, difficulty describing feelings, and externally‐oriented thinking. Social well‐being was investigated with the UCLA Loneliness Scale—3 items (Hughes et al. 2004) and the Lubben Social Network Scale (LSNS‐6) (Lubben et al. 2006).

Pain‐related coping strategies were administered with the revised version of the Coping Strategies Questionnaire (CSQ‐R‐I), addressing both maladaptive (catastrophism and praying) and adaptive (distraction, ignoring pain, distance from pain, and self‐affirmation) coping responses (Monticone et al. 2014). To specifically investigate the catastrophizing coping style, the Pain Catastrophizing Scale (PCS)—Italian Version was also administered, measuring feelings of helplessness, magnification, and rumination (Monticone et al. 2012). Details on psychological assessment tools are presented in Supplementary Materials.

#### 2.2.3 Personality‐Trait Assessment

Personality traits and psychopathological syndromes were identified by administering the Millon Multiaxial Clinical Scale – third version (MCMI‐III). It is a validated self‐administered inventory with 175 true‐false questions, divided into 14 clinical patterns of personality based on Axis II disorders of DSM‐IV, and ten clinical syndrome scales based on Axis I disorders of DSM‐IV. The scoring utilizes Base Rate (BR) scores, and a BR score of 75–84 is suggestive of a significant personality trait or mental health concern, deserving of clinical attention but not indicative of a pathological trait; whereas a score of 85 and higher indicates a persistent, significant clinical concern and a pathological personality trait (Zennaro et al. 2013).

#### 2.2.4 Cognitive Assessment

A subgroup of 33 patients underwent a neuropsychological test battery administered by trained professionals (A.T., E.S., A.F., V.F.). It included multiple cognitive domains, such as attention, executive function, and social cognition, based on recent literature on neuropsychological profiles of patients with chronic pain (Chaves et al. 2021; Grabli et al. 2022; Indart et al. 2017; Jacobsen et al. 2021; Ojeda et al. 2017).

To have a comprehensive but manageable cognitive assessment while minimizing participant fatigue, we also included a screening test and brief tasks that evaluate language and memory domains.

Accordingly, the Montreal Cognitive Assessment (MoCA) (Santangelo et al. 2015) was used as a measure of global cognitive functioning, for which permission had been granted for use in our research. The self‐administered cognitive function instrument (CFI) was included to account for potential subjective cognitive difficulties. Attentive and executive functions were assessed with the Stroop test (Amato et al. 2006), the version of the Hayling task of the ECAS (Poletti et al. 2016), the Multiple Features Targets Cancellation task (MFTC) (Marra et al. 2013), and the Digit Span Backward (Monaco et al. 2013). Processing speed was evaluated with the Symbol Digit Modalities test (Amato et al. 2006). For social cognition, the Story‐Based Empathy task (SET) (Dodich et al. 2015) and the Ekman‐60 Faces Test (Dodich et al. 2014) were used for social cognition. Memory was evaluated with the RMT Unknown Faces Test (Smirni et al. 2019) and with the digit span forward (Monaco et al. 2013). Language abilities were evaluated using the naming sub‐test of the Screening for Aphasia in NeuroDegeneration battery (SAND) (Catricalà et al. 2017) and the phonemic verbal fluency test (letter F, ENPA). Additionally, we included a measure of cognitive reserve, i.e., the Cognitive Reserve Index questionnaire (CRIq) (Nucci et al. 2012), to account for individual differences in vulnerability to cognitive, functional, or clinical decline (Stern 2003). Details on neuropsychological tests are described in Table S1.

### 2.3 Statistical Analysis

We compared demographic, clinical, and neuropsychological data between patients (*N* = 42) and HC (*N* = 42) groups using the chi‐squared test and the independent samples *t*‐test. When data did not follow normal distributions, Mann–Whitney *U* test and Fisher's exact test were performed. The conservative Bonferroni correction was applied to adjust our results.

To test whether the intensity of pain, the disease duration, the impact of symptoms on social life and work were related to psychological and cognitive dysfunctions, partial correlation analyses, with age and education as covariates, were performed between clinical data and uncorrected neuropsychological scores of all patients. Exploratory Pearson or Spearman correlations—depending on the normality of the data distribution—were also run between psychological and cognitive variables.

Additionally, in view of limited literature data on the neuropsychological profile of patients with persistent idiopathic facial pain (PIFP), we compared clinical and neuropsychological data between PIFP and age‐, sex‐, and education‐matched group of HCs using non‐parametric statistics. Statistical analyses were performed using *jamovi 2.4.11* (Jamovi 2022), and no analysis code was used.

## 3 Results

We included 84 individuals, 42 diagnosed with distinct chronic orofacial pain conditions and 42 age‐, sex‐ and education‐matched healthy volunteers. Demographic and clinical data are reported in Table 1 and in Tables S2 and S3.

**TABLE 1:** Summary of demographic and clinical data.

		COPc (*N* = 42)	PIFP (*N* = 23)	HC (*N* = 42)	Group contrasts	
	No.	Mean (SD)	Mean (SD)	Mean (SD)	COPc vs. HC	PIFP vs. HC
**Demographic data**						
Sex (female/male)^a^	84	33/9	17/6	31/11	Fisher exact test: *p* = 0.798	Fisher exact test: *p* = 0.993
Years of Age	84	46.6 (14.19) Min/Max: 22/76	49.61 (15.46) Min/Max: 22/69	52.5 (10.38) Min/Max: 23/72	*t* = 0.816; *p* = 0.417	*U* = 460; *p* = 0.752
Years of Education	84	14.8 (3.37) Min/Max: 8/23	15.00 (3.93) Min/Max: 8/22	14.4 (3.30) Min/Max: 8/23	*U* = 804; *p* = 0.480	*U* = 414; *p* = 0.333
**Clinical data**						
NRS at the time of evaluation (0–10)	38	3.44 (2.2.61) Min/Max: 0/9	3.38 (2.88) Min/Max: 0/9	—	—	—
Illness Duration (months)	42	107.45 (116.94) Min/Max: 8/505	67.82 (69.71) Min/Max: 8/247	—	—	—
Impact of pain on social life (0–5)	33	2.66 (1.99) Min/Max: 0/5	2.28 (2.25) Min/Max: 0/5	—	—	—
Impact of pain on work life (0–5)	33	2.39 (2.15) Min/Max: 0/5	1.65 (2.27) Min/Max: 0/5	—	—	—
Pain medications^b^ (Yes/No)^a^	40	28/12	16/7	—	—	—
Sleep impairment (Yes/No)^a^	42	20/22	10/13	—	—	—
Sleep medications (Yes/No)^a^	42	11/31	7/16	—	—	—
Previous psychiatric concerns/diagnosis (Yes/No)^a^	39	12/27	7/14	—	—	—

Abbreviations: COPc, Chronic Orofacial Pain Cohort; HC, healthy control group; PIFP, persistent idiopathic facial pain; *t*, independent sample *t*‐test; *U*, Mann–Whitney *U* test;

^a^ Values represent the number of cases per category.

^b^ The accurate description of patients’ pharmacological intake is presented in **Table** **S3**.

The specific patients’ diagnoses are presented in Table S2: 54.8% of patients had PIFP, while 14.3% and 11.9% were diagnosed with cephalalgia and tension‐type headache, respectively. Trigeminal neuralgia was present in 9.5% of patients, one patient had post‐herpetic facial pain, and another was diagnosed with burning mouth syndrome. Finally, two patients (4.8%) had facial pain not otherwise specified. For clarity, we refer to the entire group of patients with chronic orofacial pain as COPc (chronic orofacial pain cohort), while using PIFP to indicate the specific subgroup diagnosed with persistent idiopathic facial pain.

### 3.1 Psychological Profiles and Coping Strategies

Group comparisons showed that COPc patients had higher levels of anxiety and depression symptoms, lower quality of life, lower quality of mental and physical health, and poorer social well‐being compared to HCs. Moreover, COPc showed significantly higher traits of alexithymia, in particular, in identifying and describing feelings subscales than HCs (Table 2).

**TABLE 2:** Psychological profiles of COPs and HCs.

			COPc		PIFP		HC			Group contrasts	
	Range	No.	Mean (SD)	No.	Mean (SD)	No.	Mean (SD)			COPc vs. HC	PIFP vs. HC
**MOOD**											
Anxiety (HADS)	0–20	42	7.90 (5.2)	23	7.91 (5.3)	42	5.3 (3.4			***t* = −2.596; *p = *0.011**	*U* = 355.5; *p = *0.055
Depression (HADS)	0–16	42	4.59 (4.2)	23	5.04 (4.5)	42	3.3 (2.9)			*U* = 392; *p = *0.213	*U* = 431; *p = *0.213
Depression (BDI‐II)	0–39	25	13.6 (11.3)	12	15.41 (14.3)	40	6.4 (6.4)			***t* = −2.596; *p = *0.003** ^*^	*U* = 171; *p = *0.135
**QUALITY OF LIFE**											
Eurohis‐QoL 8‐item	17–40	39	27.2 (4.9)	22	27.72 (4.7)	42	31.5 (4.23)		***t* = 4.171; *p* < 0.001** ^*^		***U* = 168.5**; ***p* < 0.001)** ^*^
Physical health (SF‐12)	20–61	41	42.3 (10.5)	22	44.75 (9.5)	42	49.6 (7.2)		***t* = 3.660; *p* < 0.001** ^*^		***U* = 303; *p = *0.025**
Mental health (SF‐12)	15–66	41	43.9 (13.4)	22	42.74 (13.0)	42	52.1 (7.0)		***t* = 3.492; *p* < 0.001** ^*^		***U* = 247; *p = *0.002** ^*^
**Alexithymia**											
TAS total score	24–75	42	50.0 (11.6)	22	50.27 (12.8)	42	42.3 (9.4)		***t* = −3.302; *p = *0.001** ^*^		***U* = 294.5; *p = *0.018**
Identifying feelings (TAS subscore)	7–30	42	16.8 (6.8)	22	16.59 (7.4)	42	12.5 (5.0)		***t* = −3.258; *p = *0.002** ^*^		***U* = 3314; *p = *0.036**
Externally oriented thinking (TAS subscore)	8–27	42	18.0 (4.6)	22	18.32 (4.2)	42	17.4 (4.3)		*t* = −0.604; *p = *0.548		*U* = 411**; *p = *0**.474
Describing feelings (TAS subscore)	8–20	42	15.2 (3.5)	22	15.36 (3.7)	42	12.3 (3.0)		***t* = −3.873; *p* < 0.001** ^*^		***U* = 244; *p = *0.002** ^*^
**SOCIAL WELL‐BEING**											
Loneliness (*U*CLA‐3L)	1–9	38	4.10 (2.1)	22	4.24 (2.4)	42	2.5 (2.1)		***U* = 469; *p* < 0.001** ^*^		***U* = 260.5; *p = *0.005**
Social network size (Lubben scale)	6–28	80	16.8 (5.4)	22	16.71 (4.9)	42	19.6 (4.9)		***t* = 2.439; *p = *0.017**		***U* = 288; *p = *0.026**

*Note*: Statistically significant comparisons (*p* < 0.05) are highlighted in bold.

Abbreviations: BDI‐II, Beck Depression Index‐II; COPc, Chronic Orofacial Pain Cohort; CSQ‐R‐I, Coping Strategies Questionnaire—revised—Italian; HADS, Hospital Anxiety and Depression Scale; HC, healthy controls; PCS, Pain Catastrophizing Scale; PIFP, persistent idiopathic facial pain; SF‐12, 12‐item Short Form Survey; TAS, Toronto Alexithymia Scale; *t*, independent sample *t*‐test; *U*, Mann–Whitney *U* test; UCLA‐3L, UCLA Loneliness Scale—3 items.

* Comparisons that remain significant after Bonferroni correction are those with *p* ≤ 0.004, based on a correction for multiple comparisons (0.05/12 variables).

The assessment of coping strategies showed that COPc relied on maladaptive strategies to cope with pain, unlike HCs. In particular, patients tended to catastrophize significantly more than HCs, and they showed a heightened level of ruminative thinking and a strong feeling of hopelessness (*p* < 0.001).

**FIGURE 1:**
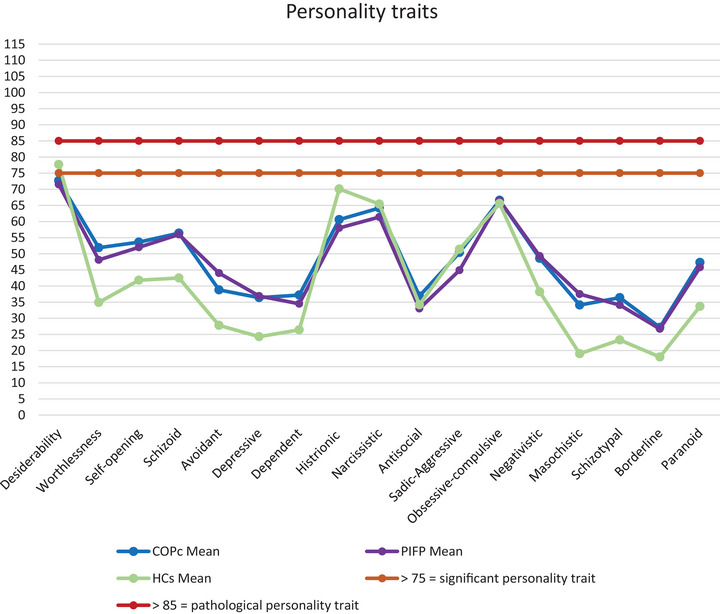
Representation of mean data distribution among the different personality traits of MCMI‐III.

**FIGURE 2:**
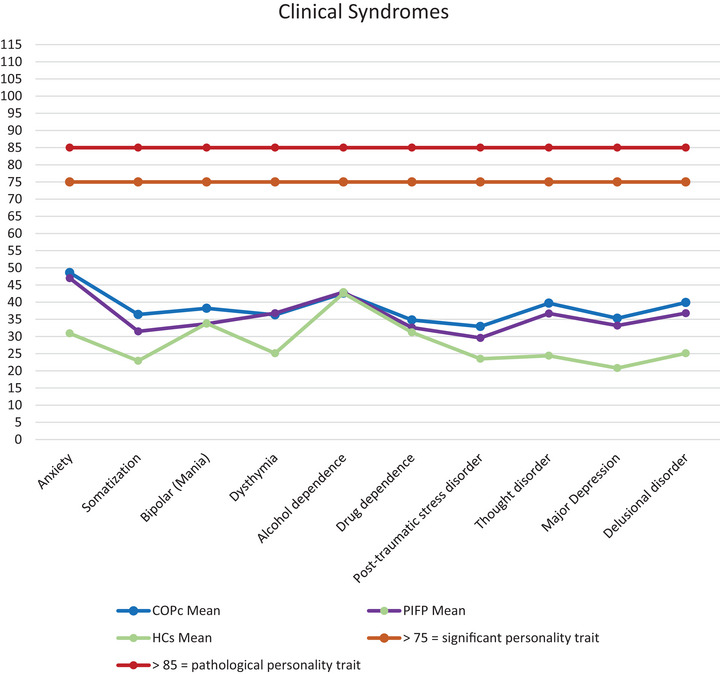
Representation of mean data of MCMI‐III clinical syndromes.

From the perspective of clinical significance, no personality trait reached the cutoffs required to determine the presence of a personality disorder (BR > 85), indicating that no trait was significantly impaired in COPc (Figure 1, Figure 2). Details are reported in Table 5.

However, group comparisons on raw scores revealed that patients had greater difficulties with self‐opening and higher levels of schizoid, masochistic, and paranoid personality traits. Notably, only the worthlessness trait was significantly higher in COPc than HCs and survived the Bonferroni correction (*p* =  0.002).

On clinical syndromes, none of the traits, either for COPc or HCs, reach the threshold for clinical significance. However, we found patients scored higher on anxiety, somatization, delusional disorder, and thought disorder than HCs, but none of these comparisons survived the Bonferroni correction.

### 3.2 Cognitive Status

Independent samples *t*‐test and Mann–Whitney *U* test showed that the scale that assesses the subjective perception of cognitive functioning is different between patients and controls, indicating that patients perceived themselves as more globally impaired at the cognitive level than the controls. Moreover, in tests evaluating specific cognitive domains, patients had a worse performance than HCs in tasks assessing social cognition, particularly the theory of mind and recognition of emotionally expressive faces, attention, and executive functions. The test measuring selective and divided attention (MFTC) showed that patients were slower than controls, although this difference does not withstand the Bonferroni correction. Notably, the only comparisons surviving Bonferroni corrections are related to attention and executive functions. In particular, patients performed significantly worse at inhibitory functions (Stroop test, errors), processing speed (symbol digit modality test), and working memory (digit span forward) tasks (Table 5).

### 3.3 Correlation Analysis

Results of the correlation analyses performed on the entire patient sample are reported in Table 6 and Tables S4 and S5. Moderate correlations (*r* or Rho between 0.33 and 0.66) were observed between psychological and cognitive variables and the measures of the impact of pain on social and work life (*p* < 0.05).

In particular, the impact of pain on social life was significantly positively correlated to some psychological dysfunctions, including maladaptive coping strategies, that is, the catastrophizing thinking and helplessness feelings, and psychopathological traits, specifically the worthlessness and self‐opening personality traits, and the anxiety, somatization, and thought disorder clinical traits. Moreover, the higher the impact of pain on social life, the lower the physical and mental health (*p* < 0.003), and the greater the feeling of having cognitive functioning difficulties (*p* = 0.028).

The impact of pain on work life, instead, showed a moderate negative correlation with the overall quality of life, and positive correlations with catastrophizing thinking, helplessness, and the sense of worthlessness (*p* < 0.05). Interestingly, pain intensity and illness duration did not show any significant correlation with the psychological and cognitive functioning, but only a weak negative correlation with the paranoid personality trait (Rho = −0.371, *p* = 0.34). Statistical details are presented in Table 6.

**FIGURE 3:**
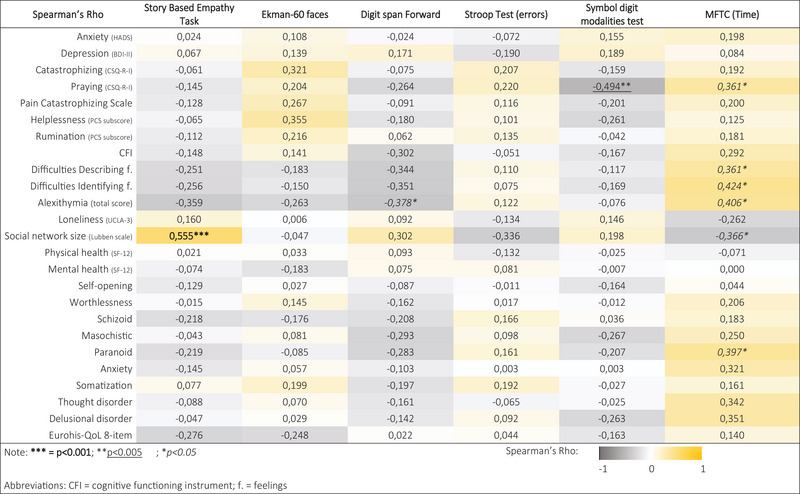
Heatmap of Rho correlation coefficients between cognitive and psychological measures that were significantly different in the pathological sample compared to the healthy control group.

When correlating psychological and cognitive variables, a significant positive correlation was found between the Story‐Based Empathy task (social cognition task) and the social network size (Lubben scale) (Rho = 0.555, *p* < 0.001). Moreover, a negative correlation was found between the symbol digit modalities task and the maladaptive coping strategy of prayer (Rho = −0.494, *p* < 0.005) and between alexithymia and two attention tasks (*p* < 0.05): the digit span forward (Rho = −0.378) and the multiple‐features target cancellation task (Rho = 406) (Figure 3).

### 3.4 The Neuropsychological Profile of PIFP

Analyses conducted on the PIFP subsample revealed a neuropsychological profile that closely overlapped with that of the overall patient cohort (Tables 2, 3, 4, 5). Group comparisons on relevant neuropsychological variables between patients diagnosed with PIFP and those with other COP conditions (based on variables that showed statistically significant differences after Bonferroni correction in the comparison between COPc and HCs) revealed a similar pattern, with no distinctive alterations (Table S6).

**TABLE 3:** Pain‐related coping strategies of COPc and HC.

			COPc		PIFP		HC	Group contrasts	
	Range	No.	Mean (SD)	No.	Mean (SD)	No.	Mean (SD)	COPc vs. HC	PIFP vs. HC
**Coping Strategies Questionnaire** (CSQ‐R‐I)									
Distraction	0–1	42	0.46 (0.27)	23	0.44 (0.25)	42	0.52 (0.27)	*t* = 0.895; *p = *0.373	*U* = 384; *p = *0.178
Distance from pain	0–1	42	0.20 (0.28)	23	0.19 (0.21)	42	0.21 (0.16)	*U* = 735; *p = *0.371	*U* = 452.5; *p = *0.668
Ignoring pain	0–1	42	0.47 (0.21)	23	0.42 (0.16)	42	0.54 (0.26)	*t* = 1.32; *p = *0.190	*U* = 359; *p = *0.089
Self‐assertiveness	0–1	42	0.66 (0.21)	23	0.62 (0.18)	42	0.69 (0.21)	*t* = 0.648; *p = *0.519	*U* = 351; *p = *0.071
Catastrophizing	0–1	42	0.47 (0.29)	23	0.46 (0.33)	42	0.14 (0.15)	***t* = −6.49**; *p* ** < 0.001** ^*^	***U* = 195.5; *p* < 0.001** ^*^
Praying	0–1	42	0.52 (0.30)	23	0.48 (0.31)	42	0.26 (0.29)	***U* = 465**; *p* **< 0.001** ^*^	***U* = 280.5; *p = *0.005** ^*^
**Pain Catastrophizing Scale** (PCS)									
Global score	0–0.8	42	0.52 (0.22)	23	0.49 (0.27)	42	0.24 (0.15)	***t* = −6.77**; *p* ** < 0.001** ^*^	***U* = 222; *p* < 0.001** ^*^
Rumination	0–1	42	0.64 (0.26)	23	0.58 (0.30)	42	0.34 (0.22)	***t* = −5.62**; *p* ** < 0.001** ^*^	***U* = 260.5; *p = *0.002** ^*^
Helplessness	0–0.91	42	0.49 (0.26)	23	0.47 (0.30)	42	0.14 (0.12)	***t* = −7.72**; *p* ** < 0.001** ^*^	***U* = 167.5; *p* < 0.001** ^*^
Magnification	0–0.75	42	0.32 (0.21)	23	0.31 (0.24)	42	0.26 (0.19)	*t* = −1.264; *p = *0.210	*U* = 420.5; *p = *0.386

*Note*: Statistically significant comparisons (*p* < 0.05) are highlighted in bold.

Abbreviations: COPc, Chronic Orofacial Pain Cohort; CSQ‐R‐I, Coping Strategies Questionnaire—revised—Italian; HC, healthy controls; PCS, pain catastrophizing scale; PIFP, persistent idiopathic facial pain; *t*, independent samples *t*‐test; *U*, Mann–Whitney *U* test.

* Comparisons that remain significant after Bonferroni correction are those with *p* ≤ 0.005, based on a correction for multiple comparisons (0.05/10 variables).

**TABLE 4:** Differences in MCMI‐III personality traits (Axis II) and MCMI‐III clinical syndromes (Axis I) among patients and HC.

			COP		PIFP		HC	Group contrasts		
		No.	Mean (SD)	No.	Mean (SD)	No.	Mean (SD)	COP vs. HC		PIFP vs. HC
Desirability		41	72.7 (15.5)	22	71.45 (17.1)	42	77.7 (10.5)	*U* = 723; *p = *0.203		*U* = 368; *p = *0.180
Worthlessness		41	51.9 (22.9)	22	48.13 (28.2)	42	34.9 (24.6)	***t* = −3.250; *p = *0.002** ^*^		***U* = 322**; *p = *0.**046**
Self‐opening		41	53.6 (18.4)	22	52.04 (20.3)	42	41.8 (19.9)	***t* = −2.788; *p = *0.007**		*U* = 351; *p = *0.118
**Personality trait (Axis II)**										
Schizoid		41	56.4 (23.8)	22	55.95 (26.2)	42	42.5 (25.1)	***U* = 592; *p = *0.014**		*U* = 329; *p = *0.060
Avoidant		41	38.8 (28.5)	22	44.04 (29.6)	42	27.8 (23.6)	*t* = −1.912; *p = *0.059		***U* = 321**; *p = *0.**047**
Depressive		41	36.4 (29.5)	22	36.90 (31.5)	42	24.3 (23.0)	*U* = 665; *p = *0.074		*U* = 378; *p = *0.234
Dependent		41	37.2 (29.2)	22	34.50 (29.8)	42	26.4 (24.6)	*t* = −1.839; *p = *0.070		*U* = 402; *p = *0.399
Histrionic		41	60.6 (25.3)	22	58.00 (27.30)	42	70.1 (21.8)	*t* = 1.829; *p = *0.071		*U* = 333; *p = *0.068
Narcissistic		41	64.2 (18.5)	22	61.36 (20.4)	42	65.4 (12.4)	*t* = 0.343; *p = *0.732		*U* = 411; *p = *0.470
Antisocial		41	36.9 (23.7)	22	33.4 (25.02)	42	34.2 (21.8)	*t* = −0.542; *p = *0.589		*U* = 436; *p = *0.718
Sadic‐Aggressive		41	50.4 (24.4)	22	44.90 (27.7)	42	51.4 (21.3)	*U* = 859; *p = *0.989		*U* = 396; *p = *0.350
Obsessive‐compulsive		41	66.6 (12.2)	22	66.54 (12.7)	42	65.6 (17.2)	*t* = −0.301; *p = *0.764		*U* = 455; *p = *0.921
Negativistic		41	48.6 (27.6)	22	49.31 (30.4)	42	38.2 (26.9)	*t* = −1.734; *p = *0.087		*U* = 353; *p = *0.125
Masochistic		41	34.1 (19.9)	22	37.50 (32.9)	42	34.1 (29.9)	***U* = 610; *p = *0.021**		*U* = 338; *p = *0.078
Schizotypal		41	36.4 (28.6)	22	34.13 (28.2)	42	23.3 (24.9)	*U* = 687; *p = *0.111		*U* = 378; *p = *0.233
Borderline		41	27.2 (24.6)	22	26.72 (25.1)	42	18 (19.6)	*U* = 662; *p = *0.070		*U* = 366; *p = *0.175
Paranoid		41	47.3 (26.5)	22	45.81 (24.2)	42	33.7 (28.6)	***U* = 638; *p = *0.042**		*U* = 342; *p = *0.089
**Clinical Syndromes (Axis I)**										
Anxiety		41	48.6 (35.8)	23	49.95 (38.1)	42	30.9 (29.6)	***t* = −2.465; *p = *0.016**		*U* = 377; *p = *0.230
Somatization		41	36.4 (29.4)	23	31.45 (29.9)	42	22.9 (25.7)	***U* = 636; *p = *0.040**		*U* = 380; *p = *0.247
Bipolar (Mania)		41	38.2 (26.4)	23	33.72 (28.5)	42	33.8 (23.7)	*U* = 776; *p = *0.437		*U* = 456; *p = *0.938
Dysthymia		41	36.3 (31.5)	23	38.81 (35.7)	42	25.1 (23.1)	*U* = 717; *p = *0.190		*U* = 414; *p = *0.496
Alcohol dependence		41	42.6 (25.5)	23	42.90 (26.3)	42	42.6 (25.4)	*U* = 837; *p = *0.830		*U* = 442; *p = *0.782
Drug dependence		41	34.8 (25.5)	23	32.59 (26.6)	42	31.2 (24.6)	*U* = 803; *p = *0.595		*U* = 456; *p = *0.938
Post‐traumatic stress disorder		41	32.9 (28.9)	23	29.59 (28.2)	42	23.5 (23.5)	*U* = 754; *p = *0.330		*U* = 429; *p = *0.640
Thought disorder		41	39.7 (27.4)	23	36.68 (30.1)	42	24.4 (24.4)	***t* = −2.680; *p = *0.009**		*U* = 373; *p = *0.209
Major depression		41	35.3 (30.6)	23	33.18 (33.6)	42	20.8 (24.0)	***U* = 620; *p = *0.028**		*U* = 366; *p = *0.175
Delusional disorder		41	39.9 (27.5)	23	36.81 (27.4)	42	25.1 (25.4)	***U* = 570; *p = *0.007**		*U* = 329; *p = *0.057

*Note*: Statistically significant comparisons (*p* < 0.05) are highlighted in bold. The BR scores’ subscales (standard deviation) are shown.

Abbreviations: BR, base rate scores of MCMI‐III scales; COPc, Chronic Orofacial Pain Cohort; HC, healthy controls; MCMI‐III, Millon multiaxial clinical inventory – third version; PIFP, persistent idiopathic facial pain; *t*, independent samples *t*‐test; *U*, Mann–Whitney *U* test.

* Comparisons that remain significant after Bonferroni correction are those with *p* ≤ 0.002, based on a correction for multiple comparisons (0.05/27 variables).

**TABLE 5:** Differences in cognitive measures (raw data) among COPs and HCs.

				COPc		PIPF		HC	Group contrasts	
	Range		No.	Mean (SD)	**No**.	Mean (SD)	**No**.	Mean (SD)	COP vs. HC	PIFP vs. HC
**Global cognitive efficiency**										
Cognitive reserve (CRI‐Q)	91–156		30	113.80 (3.04)	16	112.68 (12.66)	42	118.38 (14.82)	*t* = 1.302; *p = *0.197	*U* = 274; *p = *0.281
MoCA	15–30		30	24.33 (3.31)	16	24.69 (3.94)	42	25.59 (2.38)	*t* = 1.916; *p = *0.059	*U* = 308; *p = *0.629
Cognitive functioning instrument	0–11.5		40	3.96 (3.45)	22	3.81 (3.50)	42	2.59 (2.38)	***t* = **−**2.096; *p = *0.039**	*U* = 374; *p = *0.211
**Social cognition**										
Story‐based empathy task	3–18		33	13.90 (3.58)	17	13.37 (4.11)	41	15.68 (2.27)	***t* = 2.590; *p = *0.012**	***U* = 201; *p = *0.022**
Ekman‐60 faces	0–58		30	45.00 (10.13)	16	46.26 (5.39)	41	49.95 (3.83)	***U* = 371; *p = *0.004**	***U* = 178; *p = *0.016**
**Attention and executive functions**										
Digit span backward	2–7		33	4.27 (1.23)	17	4.12 (1.25)	41	4.68 (1.14)	*t* = 1.478; *p = *0.144	*U* = 239; *p = *0.103
Stroop test (errors)	−1–7		32	1.48 (1.81)	16	1.23 (2.06)	41	0.50 (1.24)	***U* = 405; *p = *0.003** ^*^	*U* = 222; *p = *0.085
Hayling task (ECAS)	4–12		32	9.59 (2.12)	16	8.93 (10.56)	41	10.56 (1.61)	***U* = 473; *p = *0.038**	***U* = 171; *p = *0.010**
Symbol digit modalities test	25–77		32	47.00 (14.11)	17	48.00 (13.55)	41	55.48 (96.49)	***t* = 3.065; *p = *0.003** ^*^	*U* = 218; *p = *0.052
MFTC (Accuracy)	0.47–1		32	0.952 (0.04)	16	0.95 (0.05)	41	00.930 (0.09)	*U* = 618; *p = *0.513	*U* = 260; *p = *0.369
MFTC (Time)	18–92.1		32	51.94 (18.17)	16	56.18 (18.9)	41	41.58 (11.91)	***t* = **−**2.934; *p = *0.005**	***U* = 164; *p = *0.008**
**Memory**										
Recognition memory test	10–30		32	24.22 (3.67)	16	23.60 (4.88)	41	24.22 (3.67)	*t* = 0.133; *p = *0.895	*U* = 291; *p = *0.759
Digit span forward	3–9		33	5.57 (1.03)	16	5.56 (0.89)	41	6.34 (1.10)	***U* = 415; *p = *0.002** ^*^	***U* = 194; *p = *0.012**
**Language**										
Verbal fluency	4–25	31		14.019 (5.21)	17	14.43 (5.16)	42	15.54 (3.67)	*t* = 1.395; *p = *0.167	*U* = 299; *p = *0.518
Naming (SAND)	8.5–14	33		13.51 (1.10)	17	13.44 (1.39)	40	13.83 (0.36)	*U* = 569; *p = *0.175	*U* = 275; *p = *0.254

*Note*: Statistically significant comparisons (*p* < 0.05) are highlighted in bold.

Abbreviations: COPc, Chronic Orofacial Pain Cohort; CRI‐Q, Cognitive Reserve Index questionnaire; ECAS, Edinburgh cognitive and behavioral ALS screen; HC, healthy controls; MoCA, Montreal cognitive assessment; MFTC, multiple feature test cancellation; PIFP, persistent idiopathic facial pain; SAND, screening for aphasia neurodegeneration; *t*, independent sample *t*‐test; *U*, Mann–Whitney *U* test.

* Comparisons that remain significant after Bonferroni correction are those with *p* ≤ 0.003, based on a correction for multiple comparisons (0.05/15 variables).

Compared to HCs, PIFP patients showed reduced quality of life, poorer mental health, and higher levels of alexithymia, particularly in the ability to describe their own feelings. Regarding pain coping strategies, PIFP patients were more likely to engage in catastrophizing and praying than HCs. Interestingly, no significant differences in personality traits emerged between PIFP patients and HCs after Bonferroni correction. Group differences in cognitive performance revealed poorer outcomes for PIFP patients in social cognition tasks, as well as significantly lower scores in attention, executive functioning, and short‐term memory tasks (Table 5). However, none of these comparisons remained statistically significant after Bonferroni correction (*p* ≤ 0.003).

Overall, the PIFP subsample did not reveal any additional neuropsychological traits beyond those observed in the COPc group.

## 4 Discussion

To our knowledge, this is the first study investigating the neuropsychological profile of patients with PIFP and other COP conditions with a comprehensive assessment, including psychological, personality and cognitive evaluations. We enrolled a cohort of 42 patients with COP (COPc), including 24 diagnosed with PIFP, and 42 healthy volunteers, matched for sex, age, and education. The PIFP subgroup represented the most clinically homogeneous portion of the patient sample, and it was analyzed separately to explore its specific characteristics in greater detail. Consistent with our hypothesis, we identified a compromised psychological framework across all patients, characterized by depressive symptoms, significant feelings of loneliness, alexithymia, maladaptive coping strategies, poor quality of life, and low physical and mental well‐being. This profile was also evident in patients with PIFP, who particularly exhibited psychological vulnerabilities such as difficulties in describing their own feelings, catastrophizing thinking, and poor QoL. Since no significant differences in psychological functioning emerged between patients diagnosed with PIFP and those with other COP conditions (Table S6), reflecting the comparable profiles observed across groups, the results are discussed for the entire sample.

The behavioral profile of COPc was characterized mainly by catastrophizing thinking, which negatively affects pain perception, leading to life interference and higher depression (Fillingim et al. 2011; Geisser et al. 1999; Robinson et al. 1997). Catastrophizing thinking, particularly rumination, appears to be especially prominent in COP patients (Table S7): our findings suggest that this maladaptive strategy is more frequently observed in patients with COP conditions, compared to individuals with other chronic pain conditions, such as painful peripheral neuropathy, as described in our previous study (Telesca, Soldini et al. 2024). This may indicate a distinct cognitive‐emotional profile in COPc, including PIFP, characterized by persistent negative focus on pain‐related thoughts. Literature suggests that psychopathological symptoms and maladaptive coping strategies are often associated with a poor social context (Strong and Gore 2020; VanderZee et al. 1997). Indeed, loneliness is recognized as a risk factor for poorer quality of life and cognitive decline in the elderly (Cacioppo et al. 2014; Consonni et al. 2024; Consonni, Telesca, Dalla Bella et al. 2021; Consonni, Telesca, Grazzi et al. 2021; Noguchi et al. 2023; Wilson et al. 2022). Our data supported these findings, demonstrating that reduced social network size and increased loneliness correlated with maladaptive coping strategies, particularly greater levels of catastrophizing (helplessness and rumination) with significant impact on social life (Table 6 and Table S4). Additionally, we found that patients, the whole sample, and PIFP also used prayer as a coping strategy for managing pain. Interestingly, this strategy was related to poorer performance on tasks assessing attention (Figure 3). While prayer can serve as a positive coping strategy for some individuals, in this context, it may reflect a more passive coping style, potentially contributing to cognitive strain. This interpretation is supported by supplementary correlational analyses, which revealed a significant association with catastrophizing thinking (PCS total score: *ρ* = 0.479, *p* = 0.002), suggesting increased cognitive load.

**TABLE 6:** Partial correlation coefficients (*p* values), controlling for age and education, between clinical measures and variables significantly different in COPc compared to HC.

**Variables**		NRS	Illness duration (months)	Impact of pain on social life	Impact of pain on work life
**Psychological measures**					
Anxiety (HADS)		*r* = −0.146; *p* = 0.409	*r* = −0.240; *p* = 0.146	*r* = **0.423; *p* = 0.022**	*r* = 0.202; *p* = 0.294
Depression (BDI‐II)		*r* = −0.249; *p* = 0.263	*r* = −0.067; *p* = 0.766	*r* = 0.367; *p* = 0.093	*r* = 0.256; *p* = 0.250
Eurohis‐QoL 8‐item		*r* = −0.028; *p* = 0.879	*r* = 0.218; *p* = 0.209	*r* = −0.348; *p* = 0.082	*r* = **−0.427; *p* = 0.030**
Physical health (SF‐12)		*r* = −0.002; *p* = 0.992	*r* = 0.055; *p* = 0.746	*r* = **−0.551; *p* = 0.002**	*r* = −0.329; *p* = 0.088
Mental health (SF‐12)		*r* = −0.016; *p* = 0.931	*r* = 0.293; *p* = 0.079	*r* = **−0.454; *p* = 0.015**	*r* = −0.330; *p* = 0.086
Alexithymia (TAS total score)		*r* = −0.323; *p* = 0.072	*r* = −0.263; *p* = 0.121	*r* = 0.163; *p* = 0.418	*r* = −0.106; *p* = 0.597
Identifying feelings (TAS subscore)		*r* = −0.266; *p* = 0.141	*r* = −0.185; *p* = 0.281	*r* = 0.270; *p* = 0.173	*r* = 0.055; *p* = 0.786
Describing feelings (TAS subscore)		*r* = −0.277; *p* = 0.125	*r* = −0.300; *p* = 0.075	*r* = 0.244; *p* = 0.219	*r* = −0.040; *p* = 0.845
Loneliness (UCLA‐3L)		*ρ* = −0.037; *p* = 0.839	*ρ* = 0.016; *p* = 0.929	*ρ* = 0.052; *p* = 0.797	*ρ* = 0.068; *p* = 0.736
Social network size (Lubben scale)		*r* = 0.082; *p* = 0.649	*r* = 0.194; *p* = 0.272	*r* = −0.152; *p* = 0.440	*r* = 0.084; *p* = 0.669
**Dysfunctional coping strategies**					
Catastrophizing (CSQ‐R‐I)		*r* = 0.006; *p* = 0.974	*r* = −0.009; *p* = 0.958	*r* = **0.534; *p* = 0.003**	*r* = 0.345; *p* = 0.067
Praying (CSQ‐R‐I)		*ρ* = −0.024; *p* = 0.893	*ρ* = 0.153; *p* = 0.360	*ρ* = 0.224; *p* = 0.243	ρ = 0.073 (0.707)
Pain Catastrophizing Scale (PCS)		*r* = 0.094; *p* = 0.598	*r* = 0.010; *p* = 0.953	*r* = **0.454; *p* = 0.013**	*r* = 0.343; *p* = 0.068
Rumination (PCS subscore)		*r* = 0.019; *p* = 0.915	*r* = 0.137; *p* = 0.411	*r* = 0.362; *p* = 0.054	*r* = 0.268; *p* = 0.160
Helplessness (PCS subscore)		*r* = 0.116; *p* = 0.514	*r* = −0.058; *p* = 0.729	*r* = **0.507; *p* = 0.005**	*r* = **0.393; *p* = 0.035**
**Personality traits (Axis II)**					
Worthlessness		*r* = −0.163; *p* = 0.365	*r* = −0.163; *p* = 0.365	*r* = **0.516; *p* = 0.005**	*r* = **0.381; *p* = 0.046**
Self‐opening		*r* = −0.211; *p* = 0.238	*r* = −0.211; *p* = 0.238	*r* = **0.440; *p* = 0.019**	*r* = 0.186; *p* = 0.344
Schizoid		*ρ* = 0.152; *p* = 0.398	*ρ* = 0.152; *p* = 0.398	*ρ* = 0.252; *p* = 0.195	*ρ* = 0.016; *p* = 0.935
Masochistic		*ρ* = −0.081; *p* = 0.652	*ρ* = −0.081; *p* = 0.652	*ρ* = 0.170; *p* = 0.387	*ρ* = 0.010; *p* = 0.959
Paranoid		*ρ* = **−0.371; *p* = 0.034**	*ρ* = **−0.371; *p* = 0.034**	*ρ* = −0.038; *p* = 0.848	*ρ* = −0.146; *p* = 0.459
**Clinical syndromes (Axis I)**					
Anxiety		*r* = −0.229; *p* = 0.199	*r* = −0.107; *p* = 0.528	*r* = **0.551; *p* = 0.002**	*r* = 0.270; *p* = 0.165
Somatization		*ρ* = 0.030; *p* = 0.867	*ρ* = −0.166; *p* = 0.325	*ρ* = **0.545; *p* = 0.003**	ρ = **0.435; *p* = 0.021**
Thought disorder		*r* = −0.205; *p* = 0.252)	*r* = −0.194; *p* = 0.250	*r* = **0.535; *p* = 0.003**	*r* = 0.242; *p* = 0.215
Delusional disorder		*ρ* = −0.096; *p* = 0.595	*ρ* = 0.176; *p* = 0.298	*ρ* = 0.134; *p* = 0.495	ρ = 0.176; *p* = 0.370
**Cognitive measures**					
Cognitive functioning instrument		*r* = −0.195; *p* = 0.286	*r* = −0.129; *p* = 0.453	*r* = **0.423; *p* = 0.028**	*r* = 0.271; *p* = 0.171
Story‐based empathy task		*r* = **−0.385(0.047)**	*r* = 0.172; *p* = 0.373	*r* = −0.160; *p* = 0.417	*r* = 0.090; *p* = 0.648
Emotion recognition (Ekman‐60 faces)		*ρ* = 0.007; *p* = 0.973	*ρ* = −0.177; *p* = 0.388	*ρ* = 0.333; *p* = 0.104	*ρ* = 0.297; *p* = 0.149
Inhibitory control (Stroop test—Errors)		*ρ* = 0.049; *p* = 0.814	*ρ* = **0.390; *p* = 0.040**	*ρ* = −0.078; *p* = 0.699	*ρ* = −0.078; *p* = 0.699
Information processing speed (SDMT)		*r* = −0.071; *p* = 0.729	*r* = −0.087; *p* = 0.611	*r* = −0.025; *p* = 0.902	*r* = −0.064; *p* = 0.749
Divided attention (MFTC—time)		*r* = 0.101; *p* = 0.623	*r* = −0.213; *p* = 0.276	*r* = −0.121; *p* = 0.548	*r* = −0.353; *p* = 0.071
Short‐term memory (digit span forward)		*ρ* = −0.076; *p* = 0.708	*ρ* = 0.238; *p* = 0.213	*ρ* = 0.060; *p* = 0.762	*ρ* = −0.008; *p* = 0.966

*Note*: Significant (*p* < 0.05) correlations are in bold.

Abbreviations: BDI‐II, Beck Depression Index–II; COPc, Chronic Orofacial Pain Cohort; CSQ‐R‐I, Coping Strategies Questionnaire—revised—Italian; HADS, Hospital Anxiety and Depression Scale; HC, healthy controls; MFTC, Multiple Feature Test Cancellation; PCS, Pain Catastrophizing Scale; *r*, Pearson correlation coefficient; SDMT, Symbol Digit Modalities Test; SF‐12, 12‐item short form survey; TAS, Toronto Alexithymia Scale; UCLA‐3L, UCLA Loneliness Scale—3 items; *ρ*, spearman's rank correlation coefficient.

Similar to what we previously found (Telesca, Soldini et al. 2024), COPc, including the subgroup of PIFP, showed significantly higher levels of alexithymia, meaning they had difficulties experiencing and processing their own emotions, compared to HCs. This is consistent with existing literature documenting alexithymia in orofacial pain, which has been linked to issues with emotional insight (Wise et al. 1994) and orofacial symptoms like difficulty opening the mouth, jaw movements, oro‐lingual pain, and dental pain (Kindler et al. 2019). Some studies also found a connection between alexithymia and difficulties in recognizing emotional facial expressions, suggesting these impairments are related (Roy La et al. 2021; von Piekartz et al. 2018). Our study also identified both dysfunctions in COPc, but correlation analysis showed that difficulties in identifying one's own emotions were independent of the ability to recognize others’ emotions, indicating these are separate aspects of emotional processing.

Unlike what is typically reported in chronic pain literature (Conrad et al. 2013; Naylor et al. 2017), no pathological personality traits specific to COPc emerged, except for the worthlessness trait, which distinguished COPc from HCs, but not PIFP from HCs. Nonetheless, this trait does not seem to be distinctive of COPc patients. In fact, as previously reported by Telesca, Soldini et al. (2024), worthlessness has also been observed in other clinical populations, such as those with peripheral neuropathic pain (PNP). A comparison of the severity of this trait between COPc and PNP patients (see Table S7) suggests that worthlessness may not represent a specific personality profile of COPc, but rather reflects a broader psychological response to the experience of chronic pain. Notably, in PNP, the worthlessness trait was associated with pain symptoms (Telesca, Soldini et al. 2024), but in COPc, it did not correlate with pain intensity or illness duration. Instead, the worthlessness trait was strongly correlated with other psychological variables, such as catastrophizing, mental health, quality of life, and mood alterations (*p* < 0.001) (Table S5). These results challenge the hypothesis that high pain intensity triggers psychopathological traits (Turk et al. 2016), leaving the role of personality functioning in relation to pain symptoms unclear.

Regarding cognitive functioning, our results suggest that COPc have a dysexecutive cognitive profile along with difficulties in social cognition, particularly in recognizing the emotional states of others. However, it was their performance on attention‐executive tasks that was significantly lower than that of the HC group, highlighting this domain as the most affected in COPc. A similar pattern was also observed in the PIFP subgroup, which showed impairments in the same cognitive domains; however, the comparison between PIFP and HCs did not remain statistically significant after Bonferroni correction. Few studies have investigated the cognitive profile of orofacial pain patients, revealing difficulties in attention and executive functions (Begasse de Dhaem and Robbins 2022; Coats et al. 2020), albeit inconsistently (Vuralli et al. 2018). A recent study on Burning Mouth Syndrome (BMS) patients with mild cognitive impairment (MCI) revealed more severe cognitive decline compared to a group of geriatric patients with MCI (Femminella et al. 2025). Notably, the intensity of perceived pain in BMS‐MCI patients predicted worse performance in verbal memory and attention‐switching tasks, suggesting that pain can negatively affect cognitive efficiency. Similar associations have been found between pain symptoms and cognitive dysfunction in peripheral neuropathies (Telesca, Soldini et al. 2024). Our study found only weak correlations between defective cognitive performances of COPc, pain intensity, and disease duration. This suggests that the relationship between chronic pain and cognitive functioning is likely complex and may be mediated by additional factors, including psychological and social functioning (see Figure 3).

Furthermore, given that sleep disturbances are known to negatively affect cognitive performance, we conducted additional subgroup analyses to determine whether patients reporting poor sleep quality exhibited greater cognitive impairments. As shown in Table S8, no significant differences in cognitive performance, surviving Bonferroni correction, were found between COPc patients with and without reported sleep disturbances and HCs. These findings suggest that the executive dysfunctions observed in COPc patients are not primarily driven by comorbid sleep disturbances.

We also hypothesized that psychopathological factors, such as mood disturbances, personality traits, maladaptive coping, and poor mental health, could contribute to cognitive deficits of COPc, or conversely, that impaired cognitive functioning might exacerbate these psychological difficulties. A network analysis in psychiatry showed that cognitive and psychopathological dimensions are related but independent, with symptom severity negatively correlating with cognitive performance (Chavez‐Baldini et al. 2023). As far as we know, no previous data on chronic pain exist on this topic, but our exploratory correlation analysis found that poor social network, maladaptive coping, and alexithymia are significantly related to executive dysfunctions and attentive difficulties in COPc. In contrast, anxiety and depressive symptoms were not associated with cognitive efficiency. A weak correlation was found between the paranoid personality trait and performance on an attentional task (Figure 3); however, supplementary ANCOVA analyses indicated that paranoid traits were not a significant predictor of attentional deficits (*F* = 2.69, *p* = 0.106), suggesting that personality traits do not account for the observed cognitive impairments.

An alternative hypothesis for the cognitive impairment observed in COPc may be related to the antiepileptic drugs (AED) effect. AEDs can cause memory issues, fatigue, and other behavioral changes that may include irritability, hyperactivity, or even positive psychotropic effects on mood (Coats et al. 2020; Loring et al. 2007; Tentolouris‐Piperas et al. 2018). In our sample, 54% of COPc were on AED treatment, but only 20% (i.e., 7 patients) were using AEDs as monotherapy. In all other cases, AEDs were combined with other medications (Table S3). Unfortunately, due to the heterogeneity of pharmacological treatments, we could not determine whether AEDs directly influenced cognitive deficits. Further research is needed to explore this relationship. We do not exclude the possibility that the subjective reduction in cognitive functioning and the difficulties observed in attentional tasks may be partially influenced by opioid analgesics (Block and Cianfrini 2013). Other medications, like antidepressants, seem not to significantly affect cognitive function in a sample of non‐depressed participants (Prado et al. 2018). For benzodiazepines, the findings are uncertain (Stewart 2005). However, this issue is complicated since pain and psychopathology can themselves be associated with cognitive deficits and with the extent to which patients are aware of their own cognitive problems.

An additional hypothesis concerns brain connectivity alterations underlying cognitive deficits in orofacial pain. Functional connectivity changes, particularly in the prefrontal and temporal regions, have been observed in migraine patients (Afridi et al. 2005; Begasse de Dhaem and Robbins 2022; Gil‐Gouveia et al. 2015; Moulton et al. 2011; Vuralli et al. 2018). Additionally, in BMS, structural white matter abnormalities in pain‐modulating regions can negatively affect the brain's ability to perform complex mental tasks, thereby impairing executive control and attentional performance. Unfortunately, our study lacked neuroimaging data, and future studies are needed to explore how brain connectivity alterations impact COPc.

Overall, our findings emphasize the importance of addressing cognitive domains like executive functions and attention, alongside psychological and personality factors, for a more comprehensive understanding of COPc. Indeed, to understand COPc beyond medical and clinical diagnoses, it is essential to adopt a broader, more comprehensive biopsychosocial model of pain (Gatchel et al. 2007). This model recognizes chronic pain as a complex phenomenon that arises from the simultaneous and ongoing interaction of medical, biological, environmental, and psychosocial factors. Neuropsychological functioning plays a crucial role in this context, as cognitive, emotional, and social factors can all influence the experience and management of chronic pain. A holistic approach to COP treatment must consider the interconnectedness of these domains to better address both the biological and psychosocial aspects of chronic pain, including the evaluation of economic status or employment, which would yield more concrete insights into potential interventions or support measures.

This study presents some limitations. This is the first study to employ a comprehensive neuropsychological assessment to investigate cognitive and behavioral profiles in COPc patients. However, its cross‐sectional design limits the ability to draw definitive conclusions about the neuropsychological characteristics of this population. Additionally, the sample may not be representative of the broader population of individuals with chronic orofacial pain. Notably, no patients with temporomandibular disorders, the most prevalent type of orofacial pain, were included, while the majority of the sample consisted of individuals with PIFP, a relatively rare condition. This raises concerns about the generalizability of the findings to the wider chronic facial pain population. Given the exploratory nature of the study, replication with larger samples of COP conditions, along with longitudinal follow‐up, would be valuable to confirm and extend these findings.

Moreover, the absence of a detailed description of clinical signs beyond perceived pain intensity may have hindered the identification of factors related to cognitive, psychological, and personality alterations. However, to date, there are few validated scales for assessing these aspects, and those available tend to be specific to particular COPc conditions, like headaches (Stewart et al. 2001; Yang et al. 2011). Another important limitation of this study concerns the inability to quantify in detail the effect of medication on cognitive performance. As shown in Table S3, the pharmacological classes of medications taken were highly heterogeneous, as were the combinations prescribed to each participant. Given the small number of patients taking the same type of medication in the same combination, it was not possible to perform further statistical analyses. Future studies should address this issue through an ad hoc experimental design specifically focused on dosage, pharmacological class, and their potential effects on cognitive variables.

Lastly, we included various categories of COPc, and the medical conditions of the patients in our sample are heterogeneous. However, the analysis of the PIFP subgroup, a small but homogeneous group of patients, revealed neuropsychological traits similar to those observed in the broader COPc group. Future multicenter studies involving specialists in facial disorders are needed to investigate the specific and comprehensive neuropsychological profiles associated with each condition.

To conclude, our study sheds light on the intricate relationship between COPc and cognitive, behavioral and psychological impairment, revealing that COPc is associated with significant dysexecutive altered cognitive profile, mood disorders, loneliness, emotional dysfunctions, and maladaptive coping strategies impacting social functioning and quality of life, potentially linked to pain symptoms.

Our results support the importance of a comprehensive, multidisciplinary approach to assessment and treatment. In particular, collaboration among neurologists, psychologists, dentists, physical therapists, and pain specialists is essential to address the multifaceted nature of COPc. For example, psychotherapeutic approaches may help patients manage emotional distress and maladaptive coping, while physical therapy can target musculoskeletal contributors to pain. Dental professionals can play a key role in identifying and managing orofacial triggers, and neurologists can assess for underlying neurological dysfunctions. The need for a more in‐depth assessment and investigation of neuropsychological factors and lifestyle as essential elements of a comprehensive understanding of patient functioning. This integrated model could lead to more effective, targeted therapies and ultimately improve the quality of care and outcomes for individuals with COPc.

## Author Contributions

Conceptualization: A. Telesca, M. Consonni, L. Grazzi, and D. A. Montisano. Formal analysis: A. Telesca and M. Consonni. Data curation: A. Telesca, D. A. Montisano, L. Grazzi, V. Faltracco, G. Gandini, and A. Ferrario. Funding acquisition: G. Lauria Pinter and S. Usai. Writing – original draft: A. Telesca and M. Consonni. Writing – review and editing: All authors.

## Funding

This work is suppoerted by Ricerca Corrente from the Italian Ministry of Health (RRC).

## Conflicts of Interest

The authors declare no conflicts of interest.

## Supporting information




**Supplementary Material**: brb371283‐sup‐0001‐SuppMat.docx

**Supplementary Material**: brb371283‐sup‐0002‐SuppMat.doc

## Data Availability

The data that support the findings of this study are openly available in Zenodo at https://doi.org/10.5281/zenodo.14274689.
