# Supplementary material for: Exploring Cognitive, Behavioral, and Psychological Dimensions in Persistent Idiopathic Facial Pain and Other Chronic Orofacial Pain Conditions
Source: Brain Behav. 2026 Mar 31;16(4):e71283. doi: 10.1002/brb3.71283 (PMC13112002; doi:10.1002/brb3.71283)
Supplement: Supplementary file 1 — Supplementary Material: brb371283‐sup‐0001‐SuppMat.docx [file BRB3-16-e71283-s001.docx]

Supplementary Materials

*Description of psychological assessment tools*

The Hospital Anxiety Depression Scale (HADS-A, HADS-D) (Costantini et al., 1999) consists of 14 items that evaluate anxiety and depression separately. Each item is scored from 0 to 3 using a 4-point Likert scale. The anxiety and depression subscales were computed independently. The scoring criteria indicate that a score of 0–6 points signifies no anxiety or depression, while a score of 7 or higher indicates a positive case of either condition.

To further investigate depression, we administered also the Beck Depression Inventory – II (BDI-II) (Beck et al., 1987; Sica & Ghisi, 2007). It consists of 21 items, each scored on a 4-point Likert scale (ranging from 0 to 3). A score above 13 points indicates the presence of clinical depression symptoms. For both questionnaires, a higher total score reflects a stronger clinical expression of mood traits.

Quality of life and the mental and psychological perceived well-being were assessed using the Eurohis-QoL 8-item Index (Schiavolin et al., 2015). It includes 8 items scored from 1 to 5 using a 5-point Likert scale investigating the satisfaction on different life domains (i.e., socio-economic conditions, health conditions, satisfaction with oneself and with one's relationships, etc.). A high total score corresponds to a high perception of quality of life.

The 12-Item Short Form Survey (SF-12) (Kodraliu et al., 2001), consists of 12 questions, which are divided into two main dimensions: physical health and mental health. The questions are designed to gather information on various aspects of health, such as the ability to perform daily activities, pain levels, emotional well-being, energy, and overall life satisfaction. The final scores reflect the level of disability or well-being in both areas, with higher scores indicating better quality of life.

Alexithymia (i.e.: the inability to recognize or describe one's own emotions) was assessed with the Toronto Alexithymia Scale (TAS-20) (Bressi et al., 1996): it consists of 20 items, which are grouped into three factors - Difficulty Identifying Feelings, Difficulty Describing Feelings, Externally-Oriented Thinking. Each item is rated on a 5-point Likert scale (ranging from 1 = "strongly disagree" to 5 = "strongly agree"). Higher total scores indicate a greater degree of alexithymia, with cutoff points typically used to classify individuals as having low, moderate, or high levels of alexithymia.

The UCLA Loneliness Scale - 3 items (Hughes et al., 2004) is a brief self-report measure designed to assess an individual's feelings of loneliness. Questions are rated on a 3-point Likert scale, ranging from 1 (never) to 3 (always), reflecting how often the individual experiences these feelings. Higher scores on the scale indicate greater levels of perceived loneliness (Hughes et al., 2004).

The Lubben Social Network Scale (LSNS-6) (Lubben et al., 2006) is a brief tool designed to assess the size and perceived quality of an individual's social network, particularly in terms of social support. It consists of six items that evaluate the number and closeness of relationships with family, friends, and others. Each item is rated on a 5-point Likert scale, ranging from 0 (none) to 5 (more than 9 people). Higher scores indicate a stronger, more supportive social network, while lower scores suggest greater social isolation or limited support (Lubben et al., 2006).

The Coping Strategies Questionnaire (CSQ-R-I) assess both maladaptive (catastrophism and praying) and adaptive (distraction, ignoring pain, distance from pain, and self-affirmation) coping responses. It has 27 items and each item is rated on a Likert scale from 0 (never) to 6 (always), with responses reflecting the frequency or intensity of the coping strategy used. Higher scores for certain strategies can indicate greater reliance on specific types of coping, whether adaptive or maladaptive (Monticone et al., 2014).

The Pain Catastrophizing Scale (PCS) – Italian Version was also administered, measuring feelings of helplessness, magnification, and rumination. It has 13 items, each reflecting different aspects of catastrophic thinking related to pain. Respondents rate each item on a 5-point Likert scale, ranging from 0 (not at all) to 4 (extremely), based on how frequently they experience the thought or feeling described. Higher scores indicate greater levels of pain catastrophizing (Monticone et al., 2012).

**Table S1**: List of cut off points for pathological scores* and range scores of all cognitive tests used for cognitive assessment

|  |  |  | Cut-off [min - max] | |  | |  |
| --- | --- | --- | --- | --- | --- | --- | --- |
| **GLOBAL COGNITIVE EFFICIENCY** | | | | |  | |  |
| Cognitive reserve  (CRI-Q) | | | < 69 [0 - 130] | |  | |  |
| MoCA |  |  | < 15.5 [0 - 30] | |  | |  |
| Cognitive functioning Instrument | | | The higher the score, the greater the perception of subjective cognitive decline | |  | |  |
| **SOCIAL COGNITION** | | | | |  | |  |
| Story Based Empathy Task | | | < 8.29 [0 - 18] | |  | |  |
| Ekman-60 faces | | | < 37.46 [0 - 60] | |  | |  |
| **ATTENTION AND EXECUTIVE FUNCTIONS** | | | | |  | |  |
| Digit span Backward | | | < 2.65 [0 - 9] | |  | |  |
| Stroop Test (errors) | | | < 4.24 [0 - 90] | |  | |  |
| Hayling Task (ECAS) | | |  | |  | |  |
| Symbol digit modalities test | | | < 37.9 [0 - 110] | |  | |  |
| MFTC (Accuracy) | | | / [0 - 1] | |  | |  |
| MFTC (Time) | | | < 135.73 | |  | |  |
| **MEMORY** | | | | |  | |  |
| Recognition Memory Test | | | < 21.58 [0 - 30] | |  | |  |
| Digit span Forward | | | < 4.26 [0 - 9] | |  | |  |
| **LANGUAGE** | | | | | |  | |
| Verbal Fluency | | | | < 5.7 [ / ] | |  | |
| Naming (SAND) | | | | < 9.96 [0 – 12] | |  | |
| Notes: CRI-Q = Cognitive Reserve Index questionnaire; MoCA = Montreal Cognitive Assessment; MFTC = Multiple feature test cancellation; SAND = Screening for Aphasia Neurodegeneration. * = cut-off scores refer to raw scores corrected for age, sex and education of each patient. | | | | | |  | |

| **Table S2**: Diagnosis Frequencies of patients | | | | | | | | | | |
| --- | --- | --- | --- | --- | --- | --- | --- | --- | --- | --- |
| **Diagnosis** | |  | | **Frequency** | | **Total %** | | | | **Cumulative %** |
| CHRONIC CEPHALALGIA |  |  |  | 6 |  | 14.3 % |  | 14.3 % | | |
| TRIGEMINAL NEVRALGIA |  |  |  | 4 |  | 9.5 % |  | 23.8 % | | |
| TENSION-TYPE HEADACHE |  |  |  | 5 |  | 11.9 % |  | 35.7 % | | |
| FACIAL PAIN NOS |  |  |  | 2 |  | 4.8 % |  | 40.5 % | | |
| PERSISTENT IDIOPATHIC FACIAL PAIN |  |  |  | 23 |  | 54.8 % |  | 95.2 % | | |
| POST-HERPETIC FACIAL PAIN |  |  |  | 1 |  | 2.4 % |  | 97.6 % | | |
| BURNING MOUTH SYNDROME |  |  |  | 1 |  | 2.4 % |  | 100.0 % | | |
| NOS = not otherwise specified | | | | | | | | |  | |
|  | | | | | | | | | | |

| **Table S3:** The table shows the different medication combinations taken by the patients included in this study, along with their frequency. | |
| --- | --- |
| **FREQUENCY** | **PHARMACOLOGICAL CLASS** |
| **1** | **AED + NSAIDs AB** |
| **5** | **TCA + AED** |
| **8** | **AED** |
| 2 | BDZ |
| 1 | TCA + ANALGESIC DRUG AB |
| 1 | TCA + SNRI |
| 1 | BDZ + TCA |
| 1 | TRIPTANS + ANALGESIC DRUG AB |
| **1** | **AED + OPIOIDS** |
| 1 | SNRI |
| 1 | B-BLOCKERS + NSAIDs + TRIPTANS |
| **1** | **BDZ + AED + SNRI** |
| 1 | TCA |
| 1 | TRIPTANS + ANTIDEPRESSANTS (NOS) |
| 1 | TCA + NSAIDs |
| **1** | **SNRI + AED + ANTIPSYCHOTIC DRUG** |
| **1** | **AED + BDZ** |
| **1** | **TCA + SNRI + AED** |
| 1 | SNRI |
| **1** | **OPIOID + TCA + AED** |
| 1 | TRIPTANS + ANALGESICS |
| 2 | NO PSYCHOACTIVE DRUGS |
| 7 | N.A. |
| NOTE: AED = Antiepileptics; BDZ = Benzodiazepines; NA = Not available; NOS = not otherwise specified; NSAIDs = Non-Steroidal Anti-Inflammatory Drugs; SNRI = Serotonin-Norepinephrine Reuptake Inhibitors; TCA = Tricyclic Antidepressants. | |

| **Table S4:** Correlation analysis run on a subsample of 36 patients between maladaptive coping strategies and measures of loneliness and social network. Correlation significant for p < .05 are in **bold**. | | | |
| --- | --- | --- | --- |
|  |  | **UCLA Loneliness scale** | **Social Network Scale (LSNS)** |
| UCLA Loneliness scale | Spearman’s Rho | — |  |
|  | P value | — |  |
| Lubben Social Network Scale | Spearman’s Rho | **-0.428** | — |
|  | P value | **0.013** | — |
| Praying | Spearman’s Rho | 0.166 | -0.150 |
|  | P value | 0.347 | 0.396 |
| PCS – total score | Spearman’s Rho | **0.375** | **-0.433** |
|  | P value | **0.029** | **0.011** |
| RUMINATION | Spearman’s Rho | **0.438** | **-0.479** |
|  | P value | **0.010** | **0.004** |
| HELPLESSNESS | Spearman’s Rho | 0.269 | **-0.352** |
|  | P value | 0.124 | **0.041** |
| Catastrophizing thinking (CSQ-R-I) | Spearman’s Rho | 0.169 | **-0.345** |
|  | P value | 0.339 | **0.046** |
| CSQ-R-I = Coping strategies questionnaire – revised – Italian. | | | |

| **Table S5:** Correlation analysis run on a subsample of 37 patients between the worthlessness personality trait and other clinical and psychological significant characteristic of COPs. Correlation significant for p < .05 are in **bold** | | |
| --- | --- | --- |
|  | | **Worthlessness personality trait** |
| Pain intensity (NRS) | Rho di Spearman (p value) | -0.052 (0.760) |
| Impact of pain on social life | Rho di Spearman (p value) | **0.590 (< .001) ***** |
| Impact of pain on work life | Rho di Spearman (p value) | 0.373 (0.035) * |
| Illness duration | Rho di Spearman (p value) | -0.166 (0.299) |
| Anxiety (HADS-A) | Rho di Spearman (p value) | **0.766 (< .001)***** |
| Depression (BDI-II) | Rho di Spearman (p value) | **0.799 (< .001)***** |
| Catastrophizing (PCS) | Rho di Spearman (p value) | **0.656 (< .001)***** |
| Helplessness | Rho di Spearman (p value) | **0.650 (< .001) ***** |
| Rumination | Rho di Spearman (p value) | 0.485 (0.001)** |
| Catastrophizing (CSQ-R-I) | Rho di Spearman (p value) | **0.699 (< .001)***** |
| Alexithymia | Rho di Spearman (p value) | **0.660 (< .001)***** |
| Loneliness feelings (UCLA-3) | Rho di Spearman (p value) | 0.378 (0.021)* |
| Lubben Social Network Scale | Rho di Spearman (p value) | -0.430 (0.008)** |
| Prayer | Rho di Spearman (p value) | 0.381(0.014)* |
| SF-12 Physical Health | Rho di Spearman (p value) | -0.234 (0.142) |
| SF-12 Mental Health | Rho di Spearman (p value) | **-0.766 (< .001)***** |
| Quality of life (EuroHisQoL) | Rho di Spearman (p value) | **-0.667 (< .001)***** |
| BDI-II = Beck Depression Inventory - II; CSQ-R-I = Coping Strategies Questionnaire – Revised – Italian; HADS-A = Hospital Anxiety and Depression Scale – Anxiety; HADS-D = Hospital Anxiety and Depression Scale – Depression; NRS = Numeric Rating Scale; PCS = Pain Catastrophizing Scale; SF-12 = Short-Form-12; UCLA-3 = UCLA Loneliness Scale – 3 items. * = p < 0.05; ** = p < 0.01; *** = p < 0.001 | | |

| **Table S6**: **Comparison of neuropsychological variables between PIFP and other COP patients. Displayed variables showed significant differences (Bonferroni-corrected) between the full patient sample and healthy controls** | | | | | | |
| --- | --- | --- | --- | --- | --- | --- |
|  | No. | **PIFP** | No. | **Other COP conditions** | Mann-Whitney U test | Sig. |
| Age | 23 | 49.61 (15.46) | 19 | 51.42 (14.60) | 206.5 | 0.771 |
| Education | 23 | 15.00 (3.98) | 19 | 14.52 (4.41) | 200 | 0.645 |
| **COGNITIVE EFFICIENCY** | | | |  |  |  |
| Stroop Test (errors) | 15 | 1.23 (2.06) | 17 | 1.70 (1.59) | 100.0 | 0.301 |
| Symbol digit modalities test | 16 | 48.00 (13.55) | 16 | 46.00 (15.03) | 113.5 | 0.594 |
| Digit span Forward | 16 | 5.56 (0.89) | 17 | 5.58 (1.17) | 135.5 | 1.000 |
| **PSYCHOLOGICAL DETERMINANTS** | | | |  |  |  |
| Eurohis-QoL 8-item | 22 | 27.72 (4.76) | 17 | 26.64 (5.18) | 167.5 | 0.589 |
| Physical health (SF-12) | 22 | 44.75 (9.49) | 19 | 39.66 (11.34) | 150.5 | 0.129 |
| Mental Health (SF-12) | 22 | 42.74 (13.03) | 19 | 45.35 (14.04) | 181.5 | 0.480 |
| Depression (BDI-II) | 12 | 15.41 (14.28) | 13 | 12.07 (7.94) | 73.0 | 0.806 |
| Loneliness (UCLA-3L) | 21 | 4.24 (2.42) | 17 | 4.00 (1.80) | 164.0 | 0.675 |
| Alexithymia (TAS total score) | 22 | 50.27 (12.82) | 18 | 49.83 (10.31) | 187.0 | 0.775 |
| Identifying feelings (TAS subscore) | 22 | 16.59 (7.46) | 18 | 17.11 (6.11) | 194.0 | 0.924 |
| Describing feelings (TAS subscore) | 22 | 15.36 (3.69) | 18 | 15.00 (3.51) | 183.0 | 0.692 |
| Catastrophizing (CSQ-R-I) | 23 | 0.459 (0.32) | 19 | 0.484 (0.472) | 202.5 | 0.695 |
| Praying (CSQ-R-I) | 23 | 0.481 (0.31) | 19 | 0.576 (0.30) | 183.0 | 0.374 |
| Pain Catastrophizing Scale (PCS) | 23 | 0.490 (0.27) | 19 | 0.570 (0.15) | 188.0 | 0.448 |
| Rumination (PCS subscore) | 23 | 0.583 (0.30) | 19 | 0.716 (0.18) | 200.0 | 0.694 |
| Helplessness (PCS subscore) | 23 | 0.471 (0.30) | 19 | 0.529 (0.21) | 167.0 | 0.196 |
| **PERSONALITY TRAITS AND CLINICAL SYNDROMES (MCMI-III)** | | | |  |  |  |
| Worthlessness | 22 | 48.13 (28.24) | 19 | 56.158 (14.19) | 185 | 0.538 |
| Note: COP = Chronic Orofacial Pain; PIFP = Persistent Idiopatical Facial Pain | | | | | | |

| **Table S7: One-sample t-tests were conducted to compare COPc scores with those of a painful peripheral neuropathic pain (PPN) population, which served as the reference group. Values are expressed as means.** | | | | |
| --- | --- | --- | --- | --- |
|  | **REFERENCE VALUE OF** PPN | **OBSERVED VALUE OF COPc** | Statistic (student’s t) | P value |
| Age | 53.3 | 46.6 | -1.247 | 0.220 |
| Education | 13.7 | 14.8 | 1.712 | 0.094 |
| **COGNITIVE EFFICIENCY** | | | |  |
| Stroop Test (errors) | 1.7 | 1.5 | -0.673 | 0.506 |
| Symbol digit modalities test | 47.25 | 47.0 | -0.100 | 0.921 |
| Digit span Forward | 5.83 | 5.57 | -1.42 | 0.167 |
| **PSYCHOLOGICAL DETERMINANTS** | | | |  |
| Eurohis-QoL 8-item | 24.38 | 27.2 | 3.66 | **<0.001** |
| Physical health (SF-12) | 31.73 | 42.3 | 6.46 | **<0.001** |
| Mental Health (SF-12) | 47.85 | 43.9 | -1.86 | 0.070 |
| Alexithymia (TAS total score) | 47.11 | 50.0 | 1.61 | 0.114 |
| Identifying feelings (TAS subscore) | 15.44 | 16.8 | 1.29 | 0.206 |
| Describing feelings (TAS subscore) | 14.22 | 15.2 | 1.73 | 0.091 |
| Catastrophizing (CSQ-R-I) | 0.41 | 0.47 | 1.35 | 0.185 |
| Praying (CSQ-R-I) | 0.42 | 0.52 | 1.12 | 0.268 |
| Pain Catastrophizing Scale (PCS) | 0.45 | 0.52 | 2.17 | **0.036** |
| Rumination (PCS subscore) | 0.53 | 0.64 | 2.80 | **0.008** |
| Helplessness (PCS subscore) | 0.46 | 0.49 | 0.902 | 0.373 |
| **PERSONALITY TRAITS AND CLINICAL SYNDROMES (MCMI-III)** | | | |  |
| Worthlessness | 53.47 | 51.9 | -0.451 | 0.654 |
| Note: COPc = Chort of Chronic Orofacial Pain; PPN = Painful Peripheral Neuropathy (Telesca et al., 2024) | | | | |

| **Table S8. Differences in cognitive measures (raw data) among COPc with and without sleep disturbances and HCs.** | | | | | | | |
| --- | --- | --- | --- | --- | --- | --- | --- |
|  | **COPc with sleep disorder (N=20)** | **No.** | **COPc without sleep disorder (N=22)** | **No.** | **HC (N=42)** | **No.** | **χ²**  (p value) |
| Age | 49.0 (14.44) | 20 | 51.7 (15.58) | 22 | 52.7 (10.34) | 42 | 0.572 (0.751) |
| Education | 15.8 (3.21) | 20 | 13.8 (4.65) | 22 | 14.5 (3.28) | 42 | 3.383 (0.184) |
| **GLOBAL COGNITIVE EFFICIENCY** | | | | | | | |
| Cognitive reserve  (CRI-Q) | 118.267  (11.44) | 15 | 109.33 (16.32) | 15 | 118.38 (14.82) | 42 | 4.086 (0.130) |
| MoCA | 24.82 (3.12) | 17 | 23.81 (3.52) | 16 | 25.59 (2.38) | 42 | 4.035 (0.133) |
| Cognitive functioning Instrument | 5.450 (3.68)**^b,d^** | 20 | 2.47 (2.49) | 20 | 2.595 (2.38) | 42 | **10.475 (0.005)** |
| **SOCIAL COGNITION** | | | | | | | |
| Story Based Empathy Task | 14.00 (3.04) | 17 | 13.81 (4.18) | 16 | 15.68 (2.27) | 41 | 5.3091 (0.070) |
| Ekman-60 faces | 45.21 (13.71) | 14 | 44.81 (5.95)**^c^** | 16 | 49.95 (3.83) | 41 | **9.871 (0.007)** |
| **ATTENTION AND EXECUTIVE FUNCTIONS** | | | | | | | |
| Digit span Backward | 4.17 (1.46) | 17 | 4.37 (0.95) | 16 | 4.68 (1.14) | 41 | 2.200  (0.333) |
| Stroop Test (errors) | 1.12 (1.37) | 16 | 1.844 (2.15)**^a^** | 16 | 0.500 (1.24) | 41 | **9.117 (0.010)** |
| Hayling Task (ECAS) | 10.11 (2.28) | 17 | 9.00 (1.81)**^b^** | 15 | 10.56 (1.61) | 41 | **8.956 (0.011)** |
| Symbol digit modalities test | 48.87 (13.51) | 16 | 45.12 (14.89)**^b^** | 16 | 55.48 (9.49) | 41 | **8.1857 (0.017)** |
| MFTC (Accuracy) | 0.94 (0.04) | 17 | 0.96 (0.04) | 15 | 0.93 (0.09) | 41 | 2.243 (0.326) |
| MFTC (Time) | 49.08 (14.64) | 17 | 55.19 (21.57) | 15 | 41.58 (11.91) | 41 | **6.204 (0.045)** |
| **MEMORY** | | | | | | | |
| Recognition Memory Test | 23.66 (4.99) | 15 | 24.50 (3.22) | 16 | 24.22 (3.67) | 41 | 0.045  (0.978) |
| Digit span Forward | 5.76 (1.09) | 17 | 5.37 (0.95)**^b^** | 16 | 6.34 (1.10) | 41 | **10.265 (0.006)** |
| **LANGUAGE** | | | | | | | |
| Verbal Fluency | 14.62 (4.63) | 16 | 13.53 (5.89) | 15 | 15.54 (3.67) | 42 | 1.543 (0.462) |
| Naming (SAND) | 13.58 (0.71) | 17 | 13.43 (1.42) | 16 | 13.83 (0.36) | 40 | 2.210 (0.331) |
| Notes: CRI-Q = Cognitive Reserve Index questionnaire; MoCA = Montreal Cognitive Assessment; MFTC = Multiple feature test cancellation; SAND = Screening for Aphasia Neurodegeneration; COPc = Chronic Orofacial Pain Cohort; HC = healthy controls. Statistically significant comparisons (p<0.05) are highlighted in bold.  The statistical significance threshold after Bonferroni correction was set at p ≤ .003, based on a correction for multiple comparisons across 15 variables (0.05/15). No significant pairwise comparisons (Dunn–Sidak corrected) were found unless explicitly addressed.  a = COP subgroup significantly different from healthy controls (HCs) at *p* < .05;  b = COP subgroup significantly different from healthy controls (HCs) at *p* < .01;  c = COP subgroup significantly different from healthy controls (HCs) at *p* = 0.005  d = COP subgroups significantly different at *p* = 0.009 | | | | | | | |

**Bibliography**

Beck, A. T., Steer, R. A., & Brown, G. K. (1987). *Beck depression inventory*. Harcourt Brace Jovanovich New York:.

Bressi, C., Taylor, G., Parker, J., Bressi, S., Brambilla, V., Aguglia, E.,…Invernizzi, G. (1996). Cross validation of the factor structure of the 20-item Toronto Alexithymia Scale: An Italian multicenter study. *41*(6), 551-559. <https://doi.org/10.1016/s0022-3999(96)00228-0>

Costantini, M., Musso, M., Viterbori, P., Bonci, F., Del Mastro, L., Garrone, O.,…Morasso, G. (1999). Detecting psychological distress in cancer patients: validity of the Italian version of the Hospital Anxiety and Depression Scale. *Supportive Care in Cancer*, *7*(3), 121-127. <https://doi.org/10.1007/s005200050241>

Hughes, M. E., Waite, L. J., Hawkley, L. C., & Cacioppo, J. T. (2004). A Short Scale for Measuring Loneliness in Large Surveys. *Research on Aging*, *26*(6), 655-672. <https://doi.org/10.1177/0164027504268574>

Kodraliu, G., Mosconi, P., Groth, N., Carmosino, G., Perilli, A., Gianicolo, E.,…Apolone, G. (2001). Subjective health status assessment: evaluation of the Italian version of the SF-12 Health Survey. Results from the MiOS Project. *Journal of Epidemiology and Biostatistics*, *6*(3), 305-316. <https://doi.org/10.1080/135952201317080715>

Lubben, J., Blozik, E., Gillmann, G., Iliffe, S., von Renteln Kruse, W., Beck, J. C., & Stuck, A. E. (2006). Performance of an abbreviated version of the Lubben Social Network Scale among three European community-dwelling older adult populations. *The Gerontologist*, *46*(4), 503-513. <https://doi.org/10.1093/geront/46.4.503>

Monticone, M., Baiardi, P., Ferrari, S., Foti, C., Mugnai, R., Pillastrini, P.,…Vanti, C. (2012). Development of the Italian version of the Pain Catastrophising Scale (PCS-I): cross-cultural adaptation, factor analysis, reliability, validity and sensitivity to change. *Quality of Life Research*, *21*(6), 1045-1050. <https://doi.org/10.1007/s11136-011-0007-4>

Monticone, M., Ferrante, S., Giorgi, I., Galandra, C., Rocca, B., & Foti, C. (2014). The 27-Item Coping Strategies Questionnaire — Revised: Confirmatory Factor Analysis, Reliability and Validity in Italian-Speaking Subjects with Chronic Pain. *Pain Research and Management*, *19*(3), 153-158. <https://doi.org/10.1155/2014/956828>

Schiavolin, S., Quintas, R., Ferroli, P., Acerbi, F., Brock, S., Cusin, A.,…Raggi, A. (2015). Quality of life measures in Italian neurosurgical patients: validity of the EUROHIS-QOL 8-item index. *Quality of Life Research*, *24*(2), 441-444. <https://doi.org/10.1007/s11136-014-0784-7>

Sica, C., & Ghisi, M. (2007). The Italian versions of the Beck Anxiety Inventory and the Beck Depression Inventory-II: Psychometric properties and discriminant power. *Leading-edge psychological tests and testing research*, 27-50.

Telesca, A., Soldini, E., Devigili, G., Cazzato, D., Dalla Bella, E., Grazzi, L.,…Consonni, M. (2024). Cognitive, behavioral, and psychological phenotypes in small fiber neuropathy: a case-control study. *Cortex*.
