# Supplementary material for: Exploring Cognitive, Behavioral, and Psychological Dimensions in Persistent Idiopathic Facial Pain and Other Chronic Orofacial Pain Conditions
Source: Brain Behav. 2026 Mar 31;16(4):e71283. doi: 10.1002/brb3.71283 (PMC13112002; doi:10.1002/brb3.71283)
Supplement: Supplementary file 2 — Supplementary Material: brb371283‐sup‐0002‐SuppMat.doc [file BRB3-16-e71283-s002.doc]

**Completed STROBE checklist for case-control studies**

This checklist was elaborated using formal items recommended for case-control studies from STROBE statement (https://www.strobe-statement.org).

|  | Item No | Recommendation | Respected | Comments and quotes | |
| --- | --- | --- | --- | --- | --- |
| **Title and abstract** | 1 | (*a*) Indicate the study’s design with a commonly used term in the title or the abstract | Yes | | Study design is indicated in the Background section of the abstract. It is also indicated in the Methods section:  “This was a mono-centric case-control study.” Page 2. |
|  |  | (*b*) Provide in the abstract an informative and balanced summary of what was done and what was found | Yes | | These information are stated in the abstract (study objective described, method and results described). Page 2 |
| Introduction | | |  | |  |
| Background/rationale | 2 | Explain the scientific background and rationale for the investigation being reported | Yes | | Rationale and existing literature are stated in the introduction section. Pages 3 and 4 |
| Objectives | 3 | State specific objectives, including any prespecified hypotheses | Yes | | A statement at the end of the introduction specifies the specific aims and objectives.  “Our study aims to identify the neuropsychological profile of COP patients through a detailed analysis of their cognitive and psychological functioning compared to age- and sex-matched healthy controls. Additionally, due to the lack of data on potential interactions between cognitive functioning and psychopathological characteristics, we conducted correlation analyses to determine whether there are associations between cognitive, psychological, behavioral and personality profiles.” Page 4 |
| Methods | | |  | |  |
| Study design | 4 | Present key elements of study design early in the paper | Yes | | Study design is stated in the first subsection of Methods: “This was a mono-centric case-control study.” Page 4  Key elements are all described in the method sections. Pages 4-7. |
| Setting | 5 | Describe the setting, locations, and relevant dates, including periods of recruitment, exposure, follow-up, and data collection | Mostly | | Setting, contexts, dates of inclusion, are fully described in the method section under “participants” headlines page 4.  “Forty-two COP patients (COPs) were consecutively recruited for the study from January 2021 until June 2024, at the Neuroalgology Unit, Fondazione IRCCS Istituto Neurologico Carlo Besta in Milan, Italy.” Page 4. |
| Participants | 6 | (*a*) Give the eligibility criteria, and the sources and methods of selection of participants | Yes | | Study population is described is the method section (“Participants” and “procedure”), as well as selection criteria. Pages 4 and 5. |
| Variables | 7 | Clearly define all outcomes, exposures, predictors, potential confounders, and effect modifiers. Give diagnostic criteria, if applicable | Yes | | Standardized variable definitions were used across method subheadings, which are presented in method section. Pages 5-7. |
| Data sources/ measurement | 8* | For each variable of interest, give sources of data and details of methods of assessment (measurement). Describe comparability of assessment methods if there is more than one group | Yes | | Data collection and measurement was the same for all variables, and is described in the methods section. Data came from two groups (patients and healthy controls).  Pages 5-7. |
| Bias | 9 | Describe any efforts to address potential sources of bias | Yes | | We tried to limit any potential bias by assigning each researcher a specific role based on their expertise, in order to reduce administration biases in the testing process. Furthermore, the analyses were conducted under the supervision of researchers who were not involved in the recruitment and assessment of the patients.  Additionally, in order to have a comprehensive but manageable cognitive assessment while minimizing participant fatigue, we also included a screening test and brief tasks that evaluate language and memory domains.  Pages 4 and 6. |
| Study size | 10 | Explain how the study size was arrived at | Yes | | The method states that the sample size was reached by consecutively recruiting patients and healthy controls from January 2021 to June 2024. Page 4 |
| Quantitative variables | 11 | Explain how quantitative variables were handled in the analyses. If applicable, describe which groupings were chosen and why | Yes | | The explanation of how quantitative variables were handled in the analyses and the definition of the experimental groups is provided from page 4 to page 7. |
| Statistical methods | 12 | (*a*) Describe all statistical methods, including those used to control for confounding | Yes | | The Methods’s subheading “Statistical analysis” explains how data were handled and analysed. Page 7. |
|  |  | (*b*) Describe any methods used to examine subgroups and interactions | Yes | | This is described in the method section. Interaction examination was not applicable. Page 6. |
|  |  | (*c*) Explain how missing data were addressed | Yes | | This is described in methods’ section. Page 6. |
|  |  | (*d*) If applicable, describe analytical methods taking account of sampling strategy | N/A | | Non applicable |
|  |  | (*e*) Describe any sensitivity analyses | N/A | | Non applicable |
| Results | | |  | |  |
| Participants | 13* | (a) Report numbers of individuals at each stage of study—eg numbers potentially eligible, examined for eligibility, confirmed eligible, included in the study, completing follow-up, and analysed | Yes | | This is described at the beginning of result section, page 7. |
|  |  | (b) Give reasons for non-participation at each stage | N/A | | Non applicable |
|  |  | (c) Consider use of a flow diagram | N/A | | Use of a flow diagram was not deemed appropriate |
| Descriptive data | 14* | (a) Give characteristics of study participants (eg demographic, clinical, social) and information on exposures and potential confounders | Yes | | Table 1 describes the included participants characteristics. |
|  |  | (b) Indicate number of participants with missing data for each variable of interest | Yes | | The total numbers of recorded data for each variable are stated in variable headline of each table. Tables 1-6. |
| Outcome data | 15* | Report numbers of outcome events or summary measures | Yes | | All numbers are reported in Tables 1-6. |
| Main results | 16 | (*a*) Give unadjusted estimates and, if applicable, confounder-adjusted estimates and their precision (eg, 95% confidence interval). Make clear which confounders were adjusted for and why they were included | Yes | | In all tables we reported mean values, standard deviations, and range values. Additionally, partial correlation analyses were performed to exclude age and education levels as possible confounders. Tables 1-6 and pages 8-9. |
|  |  | (*b*) Report category boundaries when continuous variables were categorized | Yes | | Category boundaries are displayed in tables 1-6. |
|  |  | (*c*) If relevant, consider translating estimates of relative risk into absolute risk for a meaningful time period | N/A | | N/A |
| Other analyses | 17 | Report other analyses done—eg analyses of subgroups and interactions, and sensitivity analyses | Yes | | All the main analyses are reported in the results’ chapter (pp 7-8). Supplemental analyses are presented in supplemental material (pp 4-5). |
| Discussion | | |  | |  |
| Key results | 18 | Summarise key results with reference to study objectives | Yes | | Key results are described at the beginning of discussion section (page 9). They also are summarized in the conclusion section (page 12). |
| Limitations | 19 | Discuss limitations of the study, taking into account sources of potential bias or imprecision. Discuss both direction and magnitude of any potential bias | Yes | | Description of limitations is in the discussion heading. Pages 11-12. |
| Interpretation | 20 | Give a cautious overall interpretation of results considering objectives, limitations, multiplicity of analyses, results from similar studies, and other relevant evidence | Yes | | References were added where possible, and discussed. Limitations were taken into account in the discussion. Pages 9-12. |
| Generalisability | 21 | Discuss the generalisability (external validity) of the study results | Yes | | Study results were deemed generalizable as reported in the conclusion section. Page 12. |
| Other information | | |  | |  |
| Funding | 22 | Give the source of funding and the role of the funders for the present study and, if applicable, for the original study on which the present article is based | Yes | | Funding information are displayed in title page of the manuscript and in the file “Title page”. |

*Give information separately for exposed and unexposed groups.
